# Degree of pain during labor and anthropometric determinants on birth size and mode of delivery at Alex Ekwueme Federal University Teaching Hospital, Abakaliki, Nigeria

**DOI:** 10.1097/MD.0000000000048413

**Published:** 2026-05-08

**Authors:** Godwin Chinedu Uzomba, Uchenna Ezemagu, Japheth Eze, Friday Chubuzor Egba, Justus Eze, Paul Ezeonu, Michael Olaolu

**Affiliations:** a Department of Anatomy, Faculty of Basic Medical Sciences, College of Medical Sciences, Alex Ekwueme Federal University Ndufu-Alike, Abakaliki, Ebonyi, Nigeria; b Department of Paediatrics, Alex Ekwueme Federal University Teaching Hospital, Abakaliki, Ebonyi, Nigeria; c Department of Obstetrics and Gynaecology, Ebonyi State University, Abakaliki, Ebonyi, Nigeria; d Department of Obstetrics and Gynaecology, Alex Ekwueme Federal University Teaching Hospital, Ndufu-Alike, Abakaliki, Ebonyi, Nigeria; e Department of Agriculture, Faculty of Agriculture, Alex Ekwueme Federal University Ndufu Alike, Abakaliki, Ebonyi, Nigeria.

**Keywords:** infant size, labor pain, neonates, pregnancy outcome, regression

## Abstract

The study aimed to examine the influence of degree of pain during labor and anthropometric parameters, as well as maternal sociodemographic, on birth characteristics and mode of delivery during labor. The study adopted an observational cross section involving pregnant mothers and their newborns. A total of 360 pregnant mothers, certified as healthy by an obstetrician, were recruited from the antenatal ward between May 1, 2020, and June 1, 2021. Each participant voluntarily signed an informed consent form, which was provided in their preferred local dialect. This study adopted direct standard anthropometric measurements, gynecological protocols and a questionnaire-based approach. Maternal and neonatal anthropometric features, sociodemographic factors, and the mode of delivery were assessed following the guidelines of the Institute of Medicine. Male newborns have higher birth length than their female counterparts. The mode of delivery of male neonates was strongly associated with the maternal degree of pain during labor (*χ*^2^ = 5.691; *P* = .048), and could be predicted using a statistical model (adjusted *R*^*2*^ = 0.154, odds ratio: 0.542). Degree of pain during labor significantly influences the delivery outcomes. Women that gave birth to male neonates experience a nociceptive dimension of pain, primarily due to prolonged labor. Understanding the causes of prolonged pain during labor could assist clinicians in managing deliveries more effectively and prevent complications. The findings could enhance the clinical relevance and decision-making and improve the management of operative deliveries, particularly for male neonates.

## 1. Introduction

Knowledge of maternal and neonatal anthropometric characteristics is important for predicting birth outcomes. Maternal anthropometry is an essential tool for evaluating pregnancy and delivery outcomes.^[1,2]^ The simplicity of anthropometric protocols makes them an accessible method for assessing maternal and neonatal health.^[3,4]^ Excessive maternal weight gain has significant health implications, pinpointing an increased risk of birth complications,^[5]^ often leading to cesarean delivery.^[6]^ For example, high body mass index (BMI) has been associated with prolonged first and second stages of labor,^[6]^ increased risk of labor induction,^[7]^ and obstetric anal sphincter injury.^[8]^ Furthermore, a negative childbirth experience has been reported in 5 to 10% of laboring women^[9]^ which may have long term effects on maternal health. For neonates, anthropometric features are crucial in predicting survival after delivery, and influencing mental development.^[10–12]^

The biological support a fetus receives during growth and development depends on the nourishment provided by the mother, influenced by her dietary intake and environmental factors during pregnancy.^[13]^ The outcome of a newborn is influenced by maternal age, parity, and sociodemographic factors.^[6]^ Consequently, short maternal stature and macrosomic babies are associated with increased delivery complications, which predispose them to fetal mortality and morbidity.^[7,14]^

Painful uterine contraction and anal sphincter injury^[15]^ can lead to prolong labor, leading to severe complications during delivery. Active labor begins at 4 cm of cervical dilatation, and progresses at a rate of 1 cm per hour or faster until full dilatation, sometimes lasting up to 18 hours.^[16]^ Beyond this duration, labor may become increasingly severe and painful, potentially affecting neonatal health and development. Therefore, establishing an optimal labor duration limit and standardized intervention strategies is essential in healthcare facilities.

Environmental pollution, distance to health facility, stressful occupations, marital status, poor nutrition status, harmful cultural beliefs, and substances such as alcohol and nicotine have been well-documented adverse effects on fetal growth and delivery outcomes.^[17–20]^ Women with low education attainment, underemployed, unplanned pregnancies, and poor living conditions face significant disadvantages during pregnancy.^[1,21,22]^ These factors increase their risk of complications and make them more likely to rely on traditional birth attendant rather than utilizing available antenatal care services. Conversely, women with better education levels tend to be more cautious during pregnancy and exhibit greater confidence in healthcare providers. Increasing access to education and improving hygienic conditions can enhance maternal and neonatal health outcomes.

Low-income countries experienced high rates of newborn mortality due to labor pain during delivery,^[23]^ highlighting a shortfall in achieving the United Nations Sustainable Development Goals, which aim to reduce neonatal mortality to at least 12 per 1000 live births by 2030. According to available data, neonatal mortality in Nigeria remains significantly high, exceeding 37 per 1000 live births, far surpassing the United Nations Sustainable Development Goals target.^[24]^ This high neonatal mortality rate is further exacerbated by inadequate healthcare facilities, misdiagnoses, suboptimal referral systems, and poor treatment of pregnant women by undertrained healthcare professionals or unqualified birth attendants. These factors often result in prolonged labor accompanied by severe pain, leading to increased maternal and newborn mortality. Furthermore, studies on labor pain during delivery are limited, particularly in the sub-Saharan region, and often report conflicting findings.^[15,25]^ Most studies observed that prolong labor associate with negative childbirth experience and increased fear of pregnancy.^[15,25]^

Therefore, the aim of the study was to investigate the relationship between the degree of pain during labor and mode of delivery, and determine the anthropometric determinants on birth size. Estimate mode of delivery based on degree of pain during labor and anthropometrics in a combined model for both neonatal sexes while accounting for sex.

## 2. Materials and methods

### 2.1. Study design

This study was an observation cross-sectional research design conducted among mothers (age range 18–40 years) and their newborn babies.

### 2.2. Setting

The study was conducted in the labor ward of the Department of Obtstetrics and Gynecology, Alex Ekwueme Federal University Teaching Hospital Abakaliki from May 2020 to June 2021. The hospital offers general, referral, and specialized clinical services in maternal and child care.

## 3. Methods

### 3.1. Participants

The study involved 360 expectant mothers (age: 30.41 ± 4.42 years, weight 65.28 ± 7.79 kg, body height 1.61 ± 0.05 m) and their newborn (weight 3104 ± 0.61 g, length 48.14 ± 3.28 cm). The sample size was estimated using the Jayakran and Tamoghna formula, based on 27% prevalence from a previous study of expectant mothers in the labor ward,^[26]^ with a 95% confidence interval and a 5% margin of error. A minimum sample size of 303 was estimated, with an additional 10% for attrition, bringing the total sample size to 360 expectant mothers who were regular in their antenatal visit. The local ethical committee (Alex Ekwueme Federal University Teaching Hospital Abakaliki /REC/VOL 3/2020/039) approved the research protocol in accordance with the Declaration of Helsinki (2013). Each participant voluntarily signed an informed consent form, which was provided in their preferred local dialect. Only the authors had access to the information collected from the participants and the confidentiality of the data was ensured in accordance with the Data Protection Act. The exclusion criteria included multiple deliveries, age cutoff point, congenital cases, stillbirths and referral from primary or secondary healthcare providers with known complications, in line with the purpose of this study.

### 3.2. Data collection

The study adopted direct standard anthropometric protocols to measure anthropometric features.^[7,27]^ A stadiometer with a weight scale (model RGZ-160, Jiangsu Suzhou Medical Instruments Co., Shanghai, China), and non-stretchable anthropometric tape were used for measurements, recorded to the nearest 0.1 kg for weight and 0.001 m for height. From these data, BMI and waist-hip ratio were calculated. For the weight and height measurements, the subjects were instructed to look at the Frankfurt plane and stand in an erect position on a health scale without support. Their BMI (kg/m^2^) was calculated and categorized as follows: underweight (< 18.5 kg/m^2^), normal (18.5–24.9 kg/m^2^), overweight (25.0–29.9 kg/m^2^), and obese (≥ 30 kg/m^2^).^[28,29]^ The waist and hip girths of each subject were measured using a stretch-resistant tape to the nearest 0.001 m. Waist girth was measured midway between the lowest ribs and the superior border of the iliac crests at the end of a normal exhalation. Hip circumference was measured at the widest point over the buttocks. The waist-hip-ratio was classified as low (≤ 0.80), moderate (0.81–0.85), and high (≥ 0.86).^[7,30]^

The weight and length of neonates were collected within I hour of birth using an infant weighing scale (Bassinet: Model 180, Jiangsu Suzhou Medical Instruments Co., Shanghai, China), and non-stretchable anthropometric tape. The weights were classified: as low birth (< 2500 g), normal weight (2500–4500 g), and macrosomia (> 4500 g).^[7]^ The heights of the neonates were measured from the highest point on the head to the most posterior point of the heel, ensuring that the legs were completely stretched by applying gentle pressure on the knees. The head was carefully placed on the infant scale, and the measurement was recorded to the nearest 0.001 m.^[3,7]^ Head circumference was measured to the nearest 0.001 m by placing the tape around the external occipital protuberance and the forehead, ensuring the tape was snug but not tight for accurate measurement^[7]^

A structured questionnaire was used to collect data on the sociodemographic factors of expectant mothers, including age, parity, visitation to hospital, nature of drinking water, and marital status. The degree of pain during labor was assessed using author-developed ordinal scale ranging from “1 (mild) to 10 (severe).” The scale was designed obstetricians involved in the study. Modes of delivery (i.e., spontaneous vaginal and cesarean) were assessed and recorded on a standard obstetric form by 2 research assistants, certified nurses trained in midwifery.^[31]^ The internal consistency of the instruments was assessed before data collection using cronbach alpha (α > 0.68) indicating acceptable reliability.

### 3.3. Statistical analyses

Microsoft office 2007 (Microsoft Corporation, Redmond, WA) and SPSS version 23.0 (SPSS Inc. Chicago, IL) were used to organize and analyze data. Descriptive statistics were presented as mean ± standard deviation. Normal distribution was checked via Shapiro–Wilk test, and appropriate statistics tests were selected accordingly. An independent sample *t*-test was performed to test the significant differences in maternal and newborn parameters, as well as the anthropometric parameters between male and female newborns (Table 1). Pearson Coefficient examined the relationship between maternal anthropometric characteristics and newborns weights (Table 2). Analysis of variance was used to test newborn birth categories with maternal anthropometric features (Table 3). The association between maternal anthropometric and sociodemographic characteristics and mode of delivery was analyzed using Chi-square (Table 4). Regression analysis was conducted to estimate delivery outcomes based on individual maternal anthropometric and sociodemographic characteristics. Co-linearity among the dependent variables was assessed and excluded to ensure the robustness of the analysis. Logistic regression was performed to evaluate the effects of maternal and neonatal anthropometric features, and degree of pain during labor on the mode of delivery (Table 5). Effect sizes were reported as the correlation coefficient (*r*) and coefficient determination (adjusted *R*^2^) for correlation and regression analysis, respectively. For all tests, statistical significance was set at *P* < .05

**Table 1:** Independent sample *t*-test of anthropometric parameters of male and female newborns in AE-FUTHA.

Parameters	Male			Female			*P* value
	Mean ± SD	Minimum	Maximum	Mean ± SD	Minimum	Maximum	
Weight (kg)	3125.60 ± 0.63	780.00	4800.00	3086.60 ± 0.59	1250.00	4400.00	.541
Length (cm)	48.50 ± 3.29	34.00	56.00	47.82 ± 3.25	30.00	58.00	.029*
Head Circumference (cm)	34.32 ± 2.26	23.00	41.00	34.24 ± 1.96	20.00	40.00	.572

AE-FUTHA = Alex Ekwueme Federal University Teaching Hospital Abakaliki, cm = centimetre, kg = kilogram, SD = standard deviation.

* Significance at *P* < .05.

**Table 2:** Pearson correlation of maternal anthropometric characteristics and weight of newborn in AE-FUTHA.

Maternal anthropometric characteristics	Weight of newborn			
	Male	*P* value	Female	
	*r*		*r*	*P* value
Age (yr)	−0.078	.172	0.003	.067
Weight (kg)	0.090	.299	0.130	.213
Height (m)	0.068	.389	-0.059	.772
BMI (kg/m^2^)	0.060	.116	0.180	.135
Waist circumference (cm)	0.091	.761	0.112	.229
Hip circumference (cm)	0.069	.123	0.052	.441
WHR	0.016	.901	0.126	.571

AE-FUTHA = Alex Ekwueme Federal University Teaching Hospital Abakaliki, BMI = body mass index, cm = centimetre, kg = kilogram, m = metre, WHR = waist hip ratio, yr = year.

**Table 3:** Analysis of variance of newborn birth categories according to maternal age and anthropometric features at AE-FUTHA.

Maternal parameters	Birth weight categories			*P* value
	Low birth weight (< 2500 g)	Normal birth weight (2500–3999 g)	Macrosomia (> 4000 g)	
Age (yr)	30.71 ± 4.06	30.31 ± 4.71	28.64 ± 3.74	.263
Weight (kg)	63.83 ± 8.68	65.47 ± 10.00	63.90 ± 5.17	.961
Height (m)	1.62 ± 0.05	1.61 ± 0.06	1.63 ± 0.06	.433
BMI (kg/m^2^)	24.33 ± 2.14	24.35 ± 4.25	23.99 ± 3.11	.341
Waist circumference (cm)	104.43 ± 6.19	106.79 ± 6.61	106.33 ± 5.02	.315
Hip circumference (cm)	100.57 ± 6.34	103.08 ± 5.90	102.42 ± 4.94	.211
WHR	1.04 ± 0.03	1.04 ± 0.04	1.04 ± 0.03	.937

AE-FUTHA = Alex Ekwueme Federal University Teaching Hospital Abakaliki, BMI = body mass index, cm = centimetre, kg = kilogram, m = metre, WHR = waist hip ratio, yr = year.

**Table 4:** Test of association between maternal anthropometric and sociodemographic characteristics and mode of delivery.

Maternal variables		Male				Female			
		Mode of delivery		Chi-square	*P* value	Mode of delivery		Chi-squaree	*P* value
		SVD (%)	C/S (%)			SVD (%)	C/S (%)		
Age (yr)	Early	7 (6.4)	4 (5.2)	0.112	.945	4 (3.2)	6 (6.7)	3.261	.196
	Active	89 (80.9)	63 (81.8)			104 (83.9)	66 (74.2)		
	Late	14 (12.7)	10 (13.0)			16 (12.9)	17 (19.1)		
BMI (kg/m^2^)	Normal	62 (56.4)	46 (59.7)	2.249	.325	48 (38.7)	32 (36.4)	1.554	.460
	Overweight	44 (40.0)	25 (32.5)			69 (55.6)	47 (53.4)		
	Obesity	4 (3.6)	6 (7.8)			7 (5.6)	9 (10.2)		
Parity	1	34 (30.9)	19 (24.7)	3.761	.439	19 (15.3)	14 (15.7)	0.179	.996
	2	30 (27.3)	20 (26.0)			60 (48.4)	43 (25.8)		
	3	36 (32.7)	28 (10.4)			32 (25.8)	23 (25.5)		
	4	10 (9.1)	8 (10.4)			11 (8.9)	7 (7.9)		
	5	0 (0.0)	2 (2.6)			2 (1.6)	2 (2.2)		
Degree of pain during delivery	Mild	32 (29.1)	32 (41.6)	5.691	.048	44 (35.5)	31 (34.8)	0.674	.714
	Moderate	42 (38.2)	31 (40.3)			64 (51.6)	43 (48.3)		
	Severe	36 (32.7)	14 (18.2)			16 (12.9)	15 (16.9)		
Visitation to Hospital	Regular	106 (96.4)	73 (94.8)	0.260	.604	116 (93.5)	88 (98.9)	3.635	.057
	Not regular	4 (3.6)	4 (5.2)			8 (6.5)	1 (1.1)		
Nature of drinking water	Borehole	10 (9.1)	8 (10.4)	6.018	.304	2 (1.6)	5 (5.6)	4.185	.242
	Bottle	19 (17.3)	11 (14.3)			1 (0.8)	1 (1.1)		
	River water	0 (0.0)	3 (3.9)			0 (0.0)	0 (0.0)		
	Sachet	79 (71.8)	55 (71.4)			109 (87.9)	70 (78.7)		
	Well-water	1 (0.9)	0 (0.0)			12 (9.7)	13 (14.6)		
Marital status	Married	107 (97.3)	73 (94.8)	0.765	.382	124 (100.0)	89 (100.0)	-	-
	Single	3 (2.7)	4 (5.2)			0 (0.0)	0 (0.0)		

AE-FUTHA = Alex Ekwueme Federal University Teaching Hospital Abakaliki, BMI = body mass index, C/S = cearean, kg = kilogram, m = metre, SVD = spontaneous vaginal delivery, yr = year.

**Table 5:** Binary Regression of maternal anthropometric characteristics and weight of newborn in AE-FUTHA.

Maternal anthropometric characteristics	Weight of newborn			
	B	*P* value	B	*P* value
Age (yr)	−0.016	.160	−0.001	.901
Weight (kg)	−0.008	.737	0.011	.065
Height (m)	1.596	.403	−1.016	.223
BMI (kg/m^2^)	0.040	.489	0.004	.579
Waist circumference (cm)	−0.029	.379	−0.035	.067
Hip circumference (cm)	0.041	.224	0.048	.018
WHR	3.496	.239	4.669	.006

AE-FUTHA = Alex Ekwueme Federal University Teaching Hospital Abakaliki, BMI = body mass index, cm = centimetre, kg = kilogram, m = metre, WHR = waist hip ratio, yr = year.

* significant (*P* < .05). Male (*R*^*2*^ = 0.037; *F*^2^ = 0.993; *P* = .438) Female (*R*^*2*^ = 0.079; *F*^2^ = 2.501; *P* = .017).

## 4. Results

The descriptive results of maternal and neonatal anthropometries are shown in Table 6. Paired sample *t*-test confirmed significant difference in height of male and female neonates (Table 1). Table 2 shows that male and female birth weight could not depend on maternal anthropometries. Analysis of variance confirmed no difference between low, normal and macrosomic birth weights and maternal anthropometries (*F* = 16.689; *P* = .122, *η*^2^ = 0.399). In Table 4, chi-square result shows strong association between the mode of delivery of male neonates and maternal degree of pain during delivery (*χ*^2^ = 5.691; *P* = .048).

**Table 6:** Descriptive result of maternal and newborn anthropometric parameters, and newborn Apgar scores at AE-FUTHA.

Parameters		Mean ± SD	Minimum	Maximum
Maternal	Age (yr)	30.41 ± 4.42	18.00	42.00
	Weight (kg)	65.28 ± 7.79	40.00	121.00
	Height (m)	1.61 ± 0.05	1.50	1.84
	BMI (kg/m^2^)	25.37 ± 5.13	17.78	105.00
	Waist Circumference (cm)	105.56 ± 6.04	89.00	127.00
	Hip Circumference (cm)	98.05 ± 6.78	77.00	118.00
	Waist-hip-ratio	1.08 ± 0.07	0.94	1.40
Newborn	Weight (g)	3104.80 ± 0.61	780.00	4800.00
	Length (cm)	48.14 ± 3.28	30.00	58.00
	Head Circumference (cm)	34.28 ± 3.28	20.00	41.00

AE-FUTHA = Alex Ekwueme Federal University Teaching Hospital Abakaliki, BMI = body mass index, cm = centimetre, g = gram, kg = kilogram, m = metre, SD = standard deviation, yr = year.

Table 5 shows that, among male neonates, a lower odd of the delivery outcome was observed with increasing maternal pain during labor (odds ratio = 0.542), reaching nominal statistically significant (*P* = .048). The model explained a modest proportion of the variance (*R*^2^ = 0.154; *F*^2^ = 22.689; *P* = .001)). In addition, the effects estimate was moderate within single factors, and are presented in table Table 7.

**Table 7:** Logistics regression of maternal parity, degree of pain, visit to hospital and anthropometric characteristics and mode of delivery of newborns at AE-FUTHA.

Maternal anthropometric and sociodemographic factors	Male			Female		
	B	*P* value	OR	B	*P* value	OR
Age (yr)	−0.013	.736	0.987	−0.032	.333	0.969
BMI (kg/m^2^)	0.057	.257	1.058	0.042	.249	1.043
WHR	−3.923	.172	0.020	−1.455	.505	0.233
Parity	0.070	.172	1.073	−0.090	.621	0.914
Degree of pain during labor	−0.613	.005*	0.542	0.230	.303	1.259
Visit to Hospital	−0.667	.450	0.513	2.098	.060	8.147

AE-FUTHA = Alex Ekwueme Federal University Teaching Hospital Abakaliki, BMI = body mass index, kg = kilogram, m = metre, OR = odds ratio, WHR = waist hip ratio, yr = year.

* significant (*P* < .05). Male (*R*^*2*^ = 0.154; *F*^2^ = 22.689; *P* = .001) Female (*R*^*2*^ = 0.078; *F*^2^ = 12.793; *P* = .235).

The final regression model (adjusted *R*^*2*^ = 0.154; *P* = .001) derived the following equation: mode of delivery = 1.002 sex (neonate)–0.613 degree of pain during labor + 3.222 (sex: 1 = male, 0 = female)

## 5. Discussion

This observational cross-sectional study aimed to investigate the influence of degree of pain during labor on mode of delivery, and analyze maternal anthropometric determinants on birth size. The study developed a regression model to determine the extent of degree of pain during labor and anthropometric determinants, and pinpoint key factors associated with delivery outcomes. A simple anthropometric tool was used in the evaluation and prediction of birth sizes and mode of deliveries in the hospital, providing valuable insights for clinical decision-making.^[7]^ These objectives were investigated to enhance the clinical relevance of decision-making regarding operative delivery, and improve the accuracy of predictions and optimize delivery outcomes.

The anthropometric features of a mother can influence the size of a neonate.^[2]^ The current findings showed that male newborns had greater birth length than female newborn (Table 1), indicating sexual dimorphism. This difference could be attributed to genetic factors and maternal dietary intake. For instance, presence of sex chromosomes, and maternal nutritional status during pregnancy play significant impact on fetal development, contributing to the observed sexual dimorphism in birth measurements. The current finding is consistent with the studies of Kumar et al^[32]^ and Bisai,^[33]^ who found higher values in the weight and head circumference of male newborn compared to their female counterparts.

Women who gave birth to neonates with normal birth weights in the present study had slightly higher maternal weight than those who gave birth to low birth weight or macrosomic babies. This suggests that maternal weight may moderately influence neonatal birth weight, with higher maternal weight being associated with normal birth weight. Additionally, women who gave birth to neonates with normal weight exhibited normal gestational weight, indicating a potential link between appropriate weight gain during pregnancy and favorable birth outcomes. This finding aligns with Fatemeh and Saraswathi^[34]^ and Rondo et al,^[35]^ who reported significant associations between maternal gestational weight gain, body mass index, and birth outcomes.

Several studies have also highlighted positive relationships between maternal age, parity, pregnancy weight gain, and delivery outcomes in both primiparous and multiparous women.^[36–39]^ These factors are critical determinants of neonatal health, influencing birth size, mode of delivery, and overall pregnancy outcomes. Similar to the findings of Yekta et al^[40]^ and Fatemeh and Saraswathi,^[34]^ the degree of pain during labor influenced the mode of delivery for male neonates. However, sociodemographic factors such as visitation to healthcare facilities, nature of drinking water, and marital status did not significantly influence the mode of delivery. It is worth noting that although these factors did not directly influence the mode of delivery, they remain essential for ensuring safe delivery outcomes. For instance, regular visitation to healthcare facility is crucial for maintaining the health of both the pregnant woman and the fetus, contributing to better overall pregnancy outcomes.

In Nigeria, expectant women are encouraged to visit hospital regularly through the provision of a wide range of services and logistical support. This collaborated with the study of Moterani et al,^[41]^ conducted in Brazil, that significant reduction of accessibility to healthcare facilities with risk of maternal and neonatal complications. The adverse effects of drinking water on birth outcomes have become a significant global concern. In the present study, high percentage of pregnant women take relatively clean water (sachet and bottle), free from contamination and potentially harmful substances that could lead to birth complications. In line with this, Tomasz^[42]^ emphasized that clean water plays a role in influencing amniotic fluid volume, promoting fetal well-being, eliminating toxic products, and preventing pregnancy loss.^[43]^

The support and understanding between both partners during pregnancy are crucial for maternal health and ensuring a safe childbirth. This partnership can contribute to emotional well-being, improved decision-making, and a more positive birth experience. The present study confirms that most women who gave birth via spontaneous vagina delivery were married, and received financial, psychological and moral supports from their husbands. This support likely contributed to their positive birth outcomes and overall well-being during pregnancy. In contrast, unmarried women were associated with low birth weight and other birth complications. This finding is consistent with the study by Ghasemi et al,^[44]^ which reported that unmarried status is associated with an increased risk of low birth weight and preterm birth. The lack of stable support systems may contribute to these adverse birth outcomes.

Although, maternal BMI did not determine the mode of delivery in the present study, previous studies^[45–47]^ have suggested that maternal overweight or obesity is associated with an increased likelihood of Cesarean delivery. In this study, a high rate of Cesarean deliveries was observed among overweight mothers (Table 4) who gave birth to female neonates. This finding highlights the need for targeted interventions during labor to address potential complications, such as fetal size and maternal health conditions, which may contribute to the increased likelihood of Cesarean section.

Most pregnant women in the current study were urbanized, had higher education levels, and consumed energy-dense diets and drinks high in fat and sugar. This dietary pattern may have contributed to the high prevalence of overweight and obesity during pregnancy. Poor nutritional habits, characterized by increased consumption of such diets, could contribute to rising maternal overweight. These conditions are associated with pregnancy complications, including a higher risk of Cesarean delivery, and microsomal neonates. This finding highlights the need to promote healthier eating habits and lifestyle modifications during pregnancy.

Pain during labor plays a critical physiological role by stimulating the release of endorphins and oxytocin, which provide comfort and facilitate labor progression.^[48]^ For instance, the logistic regression model indicated that degree of pain during labor, in combination with neonatal sex, accounted for 15% of the variance in the mode of delivery, while the majority of variance 85% was attributed to other factors. This suggests that pain intensity influences delivery decisions, and neonatal sex may contribute to likelihood of spontaneous vaginal delivery or Cesarean section. Although 15% may seem modest, it underscores the complexity of delivery outcomes and the need to consider multiple factors in making clinical decisions. Conversely, pain arises from overstretching of the pelvic floor, contraction of pelvic ligaments and perineal muscles, and changes within the pelvic cavity. For instance, alterations in myometrial cells during labor have been associated with increased pain intensity.^[49,50]^ These physiological changes may contribute to greater discomfort in mothers delivering male neonates, potentially influencing the choice of delivery mode and the overall labor experience.

Strong pressure on the sacral nerves root from the descending fetus during labor is a significant contributor childbirth pain. As the fetus descends through the birth canal, it exerts pressure on the sacral nerves, particularly in the lower back and perineal region.^[51]^ This pressure can cause intense pain, often described as back labor or sacral pain, and is linked to nociceptive pain pathways.^[2]^ This pain influences the choice of mode of delivery with many mothers opting for Cesarean birth particularly when expecting male neonates. This decision is often driven by concerns over fetal mortality and prolonged labor, and cultural factors that prioritized male offspring. The fear of complications during vaginal delivery, especially in prolonged labor, may lead some women to choose cesarean delivery for perceived safety. These factors highlight the complex interplay between medical considerations and sociocultural influences in determining the mode of delivery.

The study employs a prospective, population-based approach, enhancing its generalizability and provides a comprehensive understanding of factors influencing maternal and neonatal outcomes. By including a diverse sample of expectant mothers, it offers valuable insights into the relationships between labor pain intensity, maternal characteristics, and delivery outcomes, making the findings more applicable to broader populations. This approach strengthens the study’s external validity and its relevance to clinical decision-making and public health interventions. However, labor pain was assessed using a non-validated scale, which may have introduced exposure misclassification and reduced measurements precisions. In additions, key clinically relevant confounders, including gestational weight gain, gestational age and analgesia were not accounted for. These limitations may affected the observed associations, and limit the generalizability of the findings. Further studies should include validated pain assessment tools and comprehensive confounding adjustment, ideally within randomized controlled trials design, to better obtain the optimal threshold for operative delivery associated with operative delivery in this population.

## 6. Conclusion

The study provided insight on association between degree of pain during labor with delivery outcomes. Understanding the causes of pain during labor can help clinicians manage deliveries more effectively and prevent complications. Additionally, this study provides comprehensive overview of the effects of maternal anthropometric characteristics on neonatal anthropometries and delivery outcomes, which could offer valuable insights for clinical practice. The findings could enhance the clinical decision-making and improve the management of operative deliveries, particularly for male neonates.

## Acknowledgments

Our thanks go to AE-FUTHA staff, midwives and the expectant mothers who voluntarily participated in the study despite painful stress of labor.

## Author contributions

**Conceptualization:** Godwin Chinedu Uzomba, Friday Chubuzor Egba, Paul Ezeonu.

**Data curation:** Godwin Chinedu Uzomba, Japheth Eze, Justus Eze, Michael Olaolu.

**Formal analysis:** Michael Olaolu.

**Investigation:** Uchenna Ezemagu, Friday Chubuzor Egba, Justus Eze, Paul Ezeonu.

**Methodology:** Godwin Chinedu Uzomba, Uchenna Ezemagu, Friday Chubuzor Egba, Justus Eze, Paul Ezeonu.

**Software:** Godwin Chinedu Uzomba.

**Supervision:** Godwin Chinedu Uzomba, Paul Ezeonu.

**Validation:** Friday Chubuzor Egba.

**Visualization:** Michael Olaolu.

**Writing – original draft:** Uchenna Ezemagu.

**Writing – review & editing:** Justus Eze, Paul Ezeonu.
